# The Medical Profession and Societies of Chicago

**Published:** 1874-01-15

**Authors:** 


### Editorial Department.

### THE MEDICAL PROFESSION AND SOCIETIES OF CHICAGO.

THE Koran says that "The works of the incredulous are like the mirage of the plain: the thirsty man takes it for water until he draws nigh to it, and then he discovers that it is nothing."

The elevation of the standard of the medical profession should be the aim and object of every practitioner of respectability in the city; and there are probably few of them who, if questioned, would not admit that such was their purpose. Yet we believe that their incredulity as to the manner in which it is to be accomplished, is the cause of the insignificant results obtained.

Much has been said and written on the subject of improvement in medical education. Much, also, has been accomplished. But the character and standing of men of science is not solely determined by even the most elaborate course of preparation in collegiate and medical universities. If no efforts—persistent efforts—looking toward mutual improvement are made by those who have left the portals of an *alma mater*, the labor expended in fitting them for a career of usefulness is well-nigh valueless.

We cannot too strongly insist, especially at the outset of another year, upon the obligation and necessity imposed upon every member of the profession in this city, of connecting himself with one or the other of its medical societies.

Time was, when the medical schools of this city satisfied the demands of the hour. The responsibility of the Fellows of each Faculty to their corporate bodies was of itself sufficient to establish a species of jurisdiction and court of appeal.

But the time has passed when the organizations provided by medical faculties were sufficient to fill the needs of the profession. The day is spent when a physician could connect himself with a college or hospital, subscribe for one or two Eastern medical periodicals, and then confine himself to a narrow circle of professional sympathy and intercourse, surveying the toiling mass of the profession around him, very much as the early English barons, who intrenched themselves in their feudal castles, defied the assaults of the rest of the world.

There are about three hundred and fifty regular practitioners in this city; and hardly one-fifth of this number are connected with the staff of our educational and charitable organizations. Shall there arise from the remaining four-fifths a power like that of the British commoners, that shall, on the one hand, control with the power of organized professional opinion those who have long exercised such authority, and, on the other, elevate the standard of all co-workers in science? We believe not only that such shall be the case, but that the elements of such forces are already in active operation.

The medical societies of our city have been shamefully neglected. No one can deny it. Needless, to-day, to enumerate the causes which have brought this about. Some of them were efficient and remediless. Nor is it necessary to refer to those halcyon days when the old "Cook County Medical Society" was worthily supported by those who actively interested themselves in all its proceedings. We speak for the present and future.

To those who have now practiced medicine during almost a half-century, and have won for themselves the respect and esteem of their associates throughout the city and country, due allowance must be made. To many such, the burden of society-work, in addition to that already imposed upon them, may be intolerable. And yet such are the men who to-day, in New York and other cities of the East, have made the discussions of their academies and societies valuable to the world, and to the numerous journals that distribute them in a general circulation.

But we have a word for those who in years and grade are just below the seniors of the profession: who have isolated themselves from the medical societies till they know less of their genius and work than of the Sultan of Acheen. Some of this grade, it is true, are connected with medical schools and hospitals, and deserve great credit for their exertions in the societies during the past year.

Our word of warning and appeal is to those who have neglected this field. The rank and file of the profession, gentlemen, have been re-inforced, during the years expended by you in that eternal vigilance which is the price of practice, by men who have brought thither an intellectual power, an untiring industry, a fund of information, and a thirst for better things, which have been actively displayed in the Medical Societies. They are thus rapidly creating a power which shall pass on all questions of facts and ethics, and constitute a court of censorship and appeal superior to the authority heretofore exercised by other organizations. State and National societies are rapidly coming to their aid by the enactment of laws which exclude, from the former, delegates from hospitals and colleges who do not also represent local societies; and the latter are proceeding to protect themselves from the dead weight of men who desire their names to appear in the lists of members without taking the trouble to enter their halls, by the enforcement of appropriate regulations.

We do not speak in behalf of either one of the two societies of our city, to the exclusion of the other. At present the "Chicago Medical Society" is largely, if not exclusively, attended by gentlemen who are residents in the West Division of the city; the "Chicago Society of Physicians and Surgeons" by those who live in the South and North Divisions. Both are supported by active and industrious members. They are totally free from the exclusive control of any man, or set of men. The societies are republican in their constitution, and open their doors and their honors widely to every physician and surgeon who has not a proven stain upon his character. And they shall, at no distant day, speak *ex cathedra* to every man who claims respectability in the medical profession.

To each and every such man we say, with all kindness and earnestness, Are you willing to forego their advantages for another year? Is it right, either as regards yourself or your fellows? Can you conscientiously deplore that condition of affairs which permits pretenders, empirics, and irregular practitioners to wear the livery of an honorable profession, when you fail to enter the lists where, surely, these evils are to be encountered?

THE ILLINOIS STATE MEDICAL SOCIETY'S TRANSACTIONS FOR 1873—WHY THEY HAVE BEEN DELAYED.—The paragraph in the last number of THE EXAMINER, relative to the delay in the issue of our State Society's Transactions for 1873, has called forth numerous replies from the authors of papers, and other members, all expressive of their indignation and surprise at the most unjust and unwarrantable action of the Publication Committee in thus delaying the issue of the Transactions, in direct violation of the positive rules and by-law of the Society.

As the Publication Committee have not seen fit to offer any explanation for themselves, we shall take the liberty of stating some facts relative to the matter, which have come within our knowledge, and which, in justice to the Society, we think should be made public.

Those members who were in attendance at the meeting in Bloomington, in May last, are aware that a large number of valuable and interesting reports were presented to the meeting. With two or three exceptions, they were presented in writing, complete, at the time of the meeting, and were placed in the hands of the Secretary in abundant season. There was nothing, therefore, to prevent the transactions from being issued promptly on time, providing the two or three delinquents had not been waited for.

One author, who presented his report, in part verbally, not being able to complete it by the time specified in the by-law, laid it aside, supposing, of course, that it would be omitted. Learning, some weeks after, that the Transactions were still open, he immediately completed his report, and handed it to the Secretary.

It will also be remembered, by those present, that the *honored* and *efficient* Secretary, himself, presented, at the meeting, a highly important and valuable report. Unfortunately, however, this report then existed, in great part at least, only in the capacious brain of its author; and there, if anywhere, it still continued to exist, when last heard from.

For three months and more past, this report, and this only, has been lacking, to complete the volume of the transactions.

The facts speak for themselves; comment is unnecessary.

What explanation the Publication Committee will have to offer to the next meeting of the Society, for this neglect of the duty and trust committed to them, remains to be seen. F. H. D.

